# Generation of Retinal Organoids with Mature Rods and Cones from Urine-Derived Human Induced Pluripotent Stem Cells

**DOI:** 10.1155/2018/4968658

**Published:** 2018-06-13

**Authors:** Guilan Li, Bingbing Xie, Liwen He, Tiancheng Zhou, Guanjie Gao, Shengxu Liu, Guangjin Pan, Jian Ge, Fuhua Peng, Xiufeng Zhong

**Affiliations:** ^1^State Key Laboratory of Ophthalmology, Zhongshan Ophthalmic Center, Sun Yat-sen University, Guangzhou, China; ^2^South China Institute for Stem Cell Biology and Regenerative Medicine, Guangzhou Institutes of Biomedicine and Health, Chinese Academy of Sciences, Guangzhou, China; ^3^Department of Neurology, The Third Affiliated Hospital of Sun Yat-sen University, Guangzhou, China

## Abstract

Urine cells, a body trash, have been successfully reprogrammed into human induced pluripotent stem cells (U-hiPSCs) which hold a huge promise in regenerative medicine. However, it is unknown whether or to what extent U-hiPSCs can generate retinal cells so far. With a modified retinal differentiation protocol without addition of retinoic acid (RA), our study revealed that U-hiPSCs were able to differentiate towards retinal fates and form 3D retinal organoids containing laminated neural retina with all retinal cell types located in proper layer as in vivo. More importantly, U-hiPSCs generated highly mature photoreceptors with all subtypes, even red/green cone-rich photoreceptors. Our data indicated that a supplement of RA to culture medium was not necessary for maturation and specification of U-hiPSC-derived photoreceptors at least in the niche of retinal organoids. The success of retinal differentiation with U-hiPSCs provides many opportunities in cell therapy, disease modeling, and drug screening, especially in personalized medicine of retinal diseases since urine cells can be noninvasively collected from patients and their relatives.

## 1. Introduction

Retinal degenerative diseases such as retinal pigmentosa and age-related macular degeneration (AMD) are the major causes of vision loss due to cell death or functional loss of photoreceptor cells (PRCs) and/or retinal pigment epithelium (RPE) [1]. The underlying mechanisms are largely unknown because of lacking human disease model and limited diseased tissues. Hence, there is no effective treatment for these diseases so far [2]. In the past decade, human induced pluripotent stem cell (hiPSC) technology has been established through somatic cell reprogramming approach and provides a huge promise for study and treatment of these kinds of degenerative diseases since hiPSCs have a capacity to differentiate all body cells like human embryonic stem cells (hESCs) [3, 4]. Especially, compared to hESCs, derivatives from hiPSCs hold subject's personal genetic information, facilitating personalized medicine.

With rapid advancement of hiPSC technology, much progress has been acquired in retinal regeneration field with hiPSCs. Many studies have demonstrated that human pluripotent stem cells (hPSCs) (hESCs and hiPSCs) are able to differentiate into not only retinal cells including PRCs and RPE cells, but also retinal organoids with architecture under specific differentiation conditions, such as two-dimensional (2D) adherent culture, 3D suspension culture, or combined 2D and 3D cultures [5–11]. More importantly, these retinal organoids could achieve a high degree of maturation with formation of outer segment discs, functional structures of light-sensing photoreceptors, which was first reported by Zhong et al. [10]. These achievements would greatly facilitate the basic and translational studies of retinal degenerative diseases. In a molecular level, mature photoreceptors in human retina consist of three subtypes, rhodopsin + rods, L/M opsin + red/green cones, and S opsin + blue cones. The cones are responsible for color vision, and daytime vision human activities depend on more. So far, generation of red/green cone-rich photoreceptors with hPSCs was hardly reported.

Many types of somatic cells, such as skin fibroblasts, cord or peripheral blood cells, keratinocytes, hair follicle cells, adipose cells, and urine cells, have been used to do reprogramming to produce hiPSCs [12–19]. Some of them have demonstrated that they can be guided to differentiate into retinal cells, even to form retinal organoids [10, 11]. Among these somatic cells, urine cells have been regarded as a preferred source for reprogramming since they can be noninvasively and routinely collected in clinical settings without any risks. Although previous studies have shown that the urine-derived hiPSCs (U-hiPSCs) can differentiate into neurons, hepatocytes, tooth, and cardiomyocytes as well [20–23], it is still unclear whether or to what extent U-hiPSCs are able to differentiate towards a retinal cell lineage.

With a modified, multistep retinal differentiation protocol without addition of retinoic acid (RA), we differentiated U-hiPSCs into 3D retinal organoids which contained laminated neural retina with all major retinal cells located in corresponding layer as in vivo. Especially, highly mature photoreceptors with rods and cones were also acquired with expression of functional proteins and formation of rudimentary outer segment. Taking advantage of convenient, noninvasive acquisition of urine cells, our data suggested that U-hiPSCs could serve as a valuable source for retinal cell therapy, disease modeling, and drug screening in retinal degenerative diseases, especially in personalized medicine.

## 2. Materials and Methods

### 2.1. hiPSC Culture

Three U-hiPSC lines (UE017, UE022, and UC005), gifts of Professor Guangjin Pan (Chinese Academy of Sciences, China) were used in this study. They were generated from urine cells of three healthy human adults using episomal iPSC reprogramming vectors previously described by Xue et al. [24]. hiPSCs were routinely maintained on a feeder-free condition with mTeSR1 medium (Stem cell Technologies, USA) and MatriGel substrate (Corning, USA). Cells were passaged every 5–7 days on 80–90% confluency. Identifiable differentiated cells were removed before passage under inverted microscope. Pluripotent characteristics of U-hiPSCs were further confirmed in molecular and morphological levels. The cells without integration of exogenous reprogramming factors and vectors were also evaluated by RT-PCR.

### 2.2. Retinal Differentiation

Retinal differentiation was performed according to a published protocol with a slight modification [10]. Briefly, hiPSCs were dissociated into small clumps and cultured in suspension with mTeSR1 medium and 10 mM Blebbistatin for embryoid bodies (EBs) formation. From D1 to D15, hiPSCs were directed to retinal fate with neural induction medium (NIM) containing DMEM/F12 (1 : 1), 1% N2 supplement (Invitrogen, USA), 1 × minimum essential media nonessential amino acids (NEAA), 2 *μ*g/ml heparin (Sigma, USA), with gradient mTeSR1 and NIM change from 3 : 1, and 1 : 1 ratio to total NIM in the first three days of differentiation. The suspending aggregates were collected and plated onto MatriGel-coated dish on D7. From day16, culture medium was changed with retinal differentiation medium (RDM) containing DMEM/F12 (3 : 1) supplemented with 2% B27 (without vitamin A, Invitrogen), 1 × NEAA, and 1% antibiotic-antimycotic. Since the 4th week after differentiation, morphologically identifiable neural retina (NR) domains along with RPE domains were mechanically detached with Tungsten needle and cultured in suspension condition for formation of retinal organoids. For long-term culture, RDM was added with 10% fetal bovine serum (Natocor, Cordoba Argentina), 100 mM Taurine (Sigma), and 2 mM GlutaMAX (Invitrogen) since D42. Different from the previous approach, retinoic acid (RA) was not added to the culture medium throughout the whole differentiation process in this study. All three U-hiPSC lines were tested for retinal differentiation performed in at least three independent experiments. W9 after differentiation, retinal organoids with good shape (*n* > 20) were selected for long-term culture and assessment at different time points. The reproducibility of generating retinal organoids with good shape (yielding more than 10 organoids per 100 mm dishes) on W7–9 was assessed for each U-hiPSC line and each independent experiment (*N*).

### 2.3. Reverse Transcription-PCR

RNA extraction was done with TRIzol Reagent (Invitrogen) following the manufacturer's protocol. One microgram of total RNA was reverse transcribed using PrimeScript First Strand cDNA Synthesis Kit (Takara, Japan). The PCR was performed by Taq DNA polymerase premix (Takara, Japan) with 35 cycles. Cycles were run at 95°C for 30 s, at 60°C for 30 s, and at 72°C for 30 s. The information of used primers is listed in Table 1.

### 2.4. Immunohistochemistry

The differentiated cells or U-hiPSCs grown on coverslips were fixed with 4% paraformaldehyde (PFA) for 10 min and washed by 0.01 M PBS twice for further experiment. Collected 3D retinal organoids were fixed with 4% PFA for 30 min at room temperature. Fixed retinal organoids were processed with sucrose gradient dehydration from 6.25%, 12.5%, to 25% successively and embedded in OCT. These organoids were sectioned using a cryostat microtome in 12 *μ*m slices. Cells or sections incubated in blocking solution containing 10% donkey serum only or 10% donkey serum with 0.25% Triton X-100 for 1 h at room temperature. Primary antibodies were incubated at the suitable dilution in PBS overnight at 4°C. After primary antibody incubation, cells or sections were washed by 0.01 M PBS for three times and then incubated with secondary antibodies for 1 hr at room temperature. The information of used antibodies was shown in Table 2. DAPI (40,6-diamidino-2-phenylindole) was used for nuclear counterstaining (Molecular Probes, USA). Fluorescence images were acquired with an LSM 510 confocal microscope (ZEISS) or an Olympus fluorescence microscope (BX53F; Olympus).

To assess differentiation efficiency of photoreceptor subtypes in retinal organoids older than W21, we measured opsins (rhodopsin, L/M opsin, and S opsin) positive area and total presumptive outer nuclear layer (ONL) area per retinal organoid (*n* = 14) with ImageJ software, according to a method published [14]. Results represented the percent area of the total ONL of the organoids. Organoids with rod-rich or cone-rich photoreceptors were defined as more than 50% rhodopsin+ or L/M opsin + area in ONL, respectively.

### 2.5. TEM Ultrastructural Analysis

Retinal organoids were fixed in a cold EM fixative (2.5% glutaraldehyde/2% PFA) overnight at 4°C. The fixed organoids were sent to TEM core facility for dehydration, embedding, sectioning, and staining in Zhongshan School of Medicine, Sun Yat-sen University. The samples were observed and imaged by transmission electron microscope (Tecnai G2 Spirit, FEI Inc.).

## 3. Results

### 3.1. The Characteristics of U-hiPSCs

**Figure 1:**
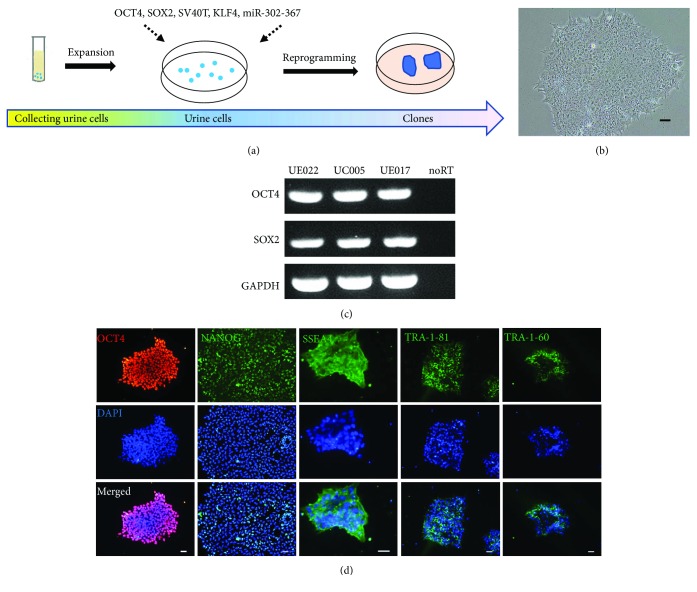
Characteristics of urine-hiPSCs. (a) The schematic of reprogramming process of integration-free hiPSCs from urine cells. (b) Representative phase contrast photograph of U-hiPSCs. Scale bar = 250 *μ*m. (c) RT-PCR showed that U-hiPSCs from all three lines expressed pluripotency markers OCT4 and SOX2. noRT: negative control without reserve transcriptase. (d) Example images of immunofluorescence staining of U-hiPSCs with pluripotency markers OCT4, NANOG, SSEA4, TRA-1-81, and TRA-1-60. Scale bars = 20 *μ*m.

For the safe application of hiPSC derivatives in future clinical settings, here, we chose three nonintegrating hiPSCs derived from urine cells to explore their capacity of retinal differentiation. The urine cells collected from healthy adults were expanded and reprogrammed by two episomal plasmids containing OCT4, SOX2, KLF4, SV40T, and miR-302-367 cluster on feeder-free and serum-free culture conditions, acquired pluripotent features like embryonic stem cells (Figure 1(a)). While cultured on MatriGel-coated surface with mTeSR1, the U-hiPSCs grew in clone with a clear boundary, exhibited a typical human PSC morphology with a high nuclear to cytoplasmic ratio, and expressed pluripotency markers OCT4, NANOG, SOX2, SSEA4, TRA-1-60, and TRA-1-81 revealed by immunofluorescence staining and/or RT-PCR (Figures 1(b)–1(d)). In addition, RT-PCR further confirmed that the U-hiPSCs within passage 20–30 used in this study did not express exogenous genes (OCT4, SOX2, KLF4, SV40T, ORIP, EBNA-1, and miR-302-367) which were forced to express during reprogramming process [Suppl.  Figure 1].

### 3.2. Induction of U-hiPSCs towards Retinal Fates

**Figure 2:**
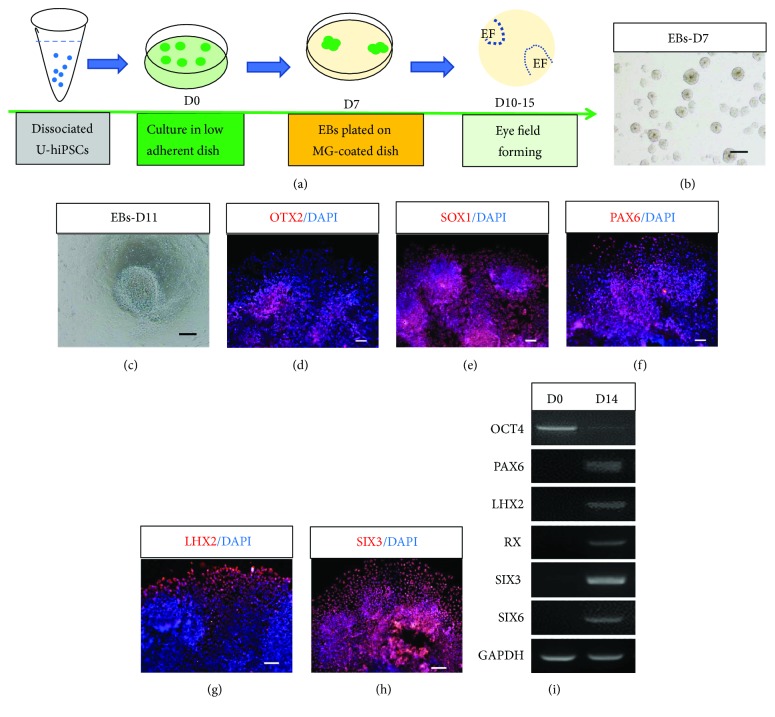
Induction of U-hiPSCs into retinal fates. (a) Schematic overview of retinal differentiation protocol from U-hiPSCs. (b) U-hiPSCs were dissociated into small clumps and formed EBs under suspension culture on D7. Scale bar = 250 *μ*m. (c) Cells spread out from EBs on adherent surface on D11. Scale bar = 250 *μ*m. (d–h) Immunofluorescence staining revealed that differentiated cells expressed markers specific for anterior neural plate and eye field cells, SOX1 (d), PAX6 (e), OTX2 (f), LHX2 (g), and SIX3 (h). Scale bars = 50 *μ*m. (i) RT-PCR showed that differentiated cells expressed EF transcription factors PAX6, LHX2, RX, SIX3, and SIX6 on D14 with low expression of pluripotency marker OCT4.

Eye development begins with formation of eye field (EF) in vivo. Here, we differentiated U-hiPSCs into the EF (Figure 2(a)). On 6 to 7 days after differentiation, cell aggregates, also called EBs (Figure 2(b)), reminiscent of competent blastomere at embryogenesis, were plated on MatriGel-coated dishes for further differentiation. Under adherent conditions, cells spread out (Figure 2(c)) and gradually acquired anterior neural fate and eye field fate two weeks after differentiation. Immunocytochemistry showed that these cells expressed the corresponding markers, such as SOX1, PAX6, OTX2, LHX2, and SIX3 (Figures 2(d)–2(h)). The reverse transcription-PCR also demonstrated that differentiated cells expressed EF transcription factors (EFTFs), PAX6, LHX2, RX, SIX3, and SIX6 on D14 with negative or low expression of pluripotency marker OCT4 (Figure 2(i)). All three U-hiPSCs (UE017, UE022, and UC005) could repeatedly differentiate towards retinal fates following the similar time course. At this early stage of retinal differentiation, no major morphological difference was observed among lines.

### 3.3. Formation of Retinal Organoids from U-hiPSCs

**Figure 3:**
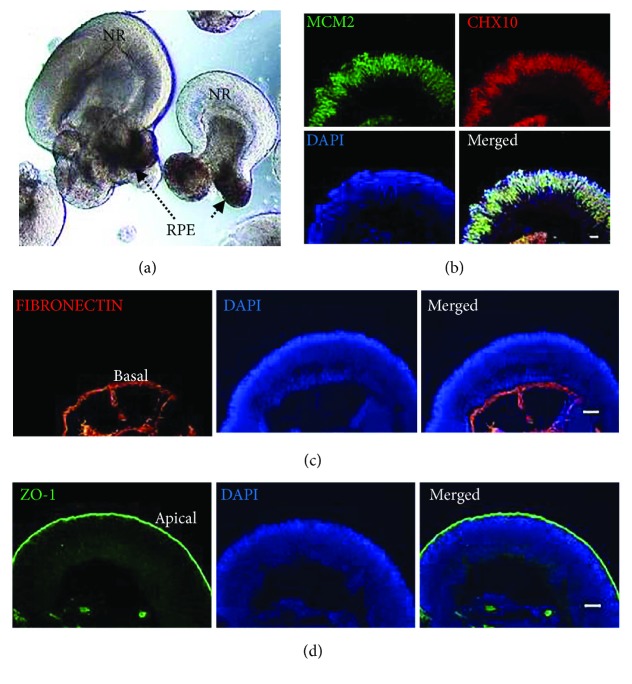
Generation and characterization of 3D retinal organoids from U-hiPSCs. (a) Example image of retinal organoids with neural retina (NR) and retinal pigment epithelium (RPE) from UE017-hiPSCs on W6 after differentiation. Scale bar = 100 *μ*m. (b) Neural retina comprised mostly retinal progenitor cells expressing CHX10 and cell proliferative marker MCM2 on W6 after differentiation. Scale bars = 50 *μ*m. (c, d) The polarity of NRs with FIBRONECTIN^+^ cells (c) located at the basal side while a tight junction marker ZO-1^+^ cells (d) at the apical side. Scale bars = 50 *μ*m.

As differentiation progressed, horseshoe-dome shapes appeared 4 weeks after differentiation. These domains were mechanically detached and collected for further culture in suspension from week 4 (W4). The retinal organoids self-formed soon after suspension culture. They consisted of a thick and continuous semitransparent neural retina (NR) attached with a roll-up RPE ball (Figure 3(a)). NRs in these organoids showed pseudostratified neural epithelium feature with typical polarity. The neural retinal epithelium was positive for retinal progenitor marker CHX10, also called VSX2, which coexpressed with proliferative marker MCM2 (Figure 3(b)). The FIBRONECTIN-positive cells accumulated at the basal side while a tight junction marker, ZO-1 positive cells, lied on the apical side (Figures 3(c) and 3(d)). All three U-hiPSCs (UE017, UE022, and UC005) generated 3D retinal organoids with at least three independent experiments per line performed, although variation in reproducibility among them existed. Of all differentiations, 72% (*N* = 11 differentiations, UE017), 67% (*N* = 3 differentiations, UE022), and 80% (*N* = 5 differentiations, UC005) of the differentiations were successful, respectively, which was similar to those from other somatic source-derived hiPSCs or hESCs [13].

### 3.4. Retinal Cell Specification and Lamination in Retinal Organoids Derived from U-hiPSCs

**Figure 4:**
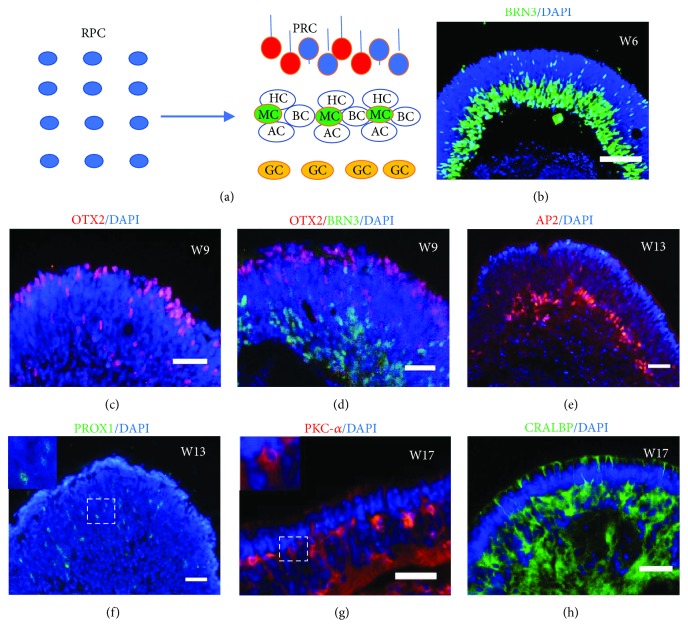
Specification and lamination of neural retina in retinal organoids derived from U-hiPSCs. (a) Schematic diagram of laminated neural retina with five retinal neurons and one glia generated from retinal progenitor cells (RPC) in vivo. PRC, photoreceptor cell; GC, ganglion cell; AC, amacrine cell; HC, horizontal cell; BC, bipolar cell; MC, Muller glial cell. (b–h) Immunofluorescence staining on retinal organoids showed that urine-derived RPCs sequentially differentiated into BRN3-positive GCs (b, d), OTX2-positive PRCs (c, d), then AP2-positive ACs(e), PROX1-positive HCs (f) (insert, higher magnification of the squared area), PKC-*α*-positive BCs (g) (insert, higher magnification of the squared area), and CRALBP-positive MCs (h) at different time windows after differentiation. All these retinal cells migrated to corresponding layers. Scale bars = 20 *μ*m.

Developing vertebrate retinas comprise five neurons and one glia. They are all specified from multipotent retinal progenitor cells (RPCs) during retina development in an ordered fashion that retinal ganglion cells (GC) are born first, followed by photoreceptor cells (PRC), amacrine cells (AC), horizontal cells (HC), and lastly by bipolar cells (BC) and Muller glial cells (MC) (Figure 4(a)) [10, 25]. Following the similar developmental rules as in vivo, urine-derived RPCs differentiated into all retinal cell lineages within the urine-derived 3D retinal organoids providing a suitable microenvironment niche. With a modified protocol without addition of RA, a key neural inducer widely used in directed retinal differentiation approaches [8–10, 13, 26, 27], most retinal organoids from U-hiPSCs grew and kept nice structure under suspension culture in low adherent dishes by changing medium three times a week. In addition, urine-derived RPCs first generated BRN3^+^ GCs as early as week five after differentiation. They gradually increased in cell number and formed a distinct layer at the basal-most zone of the neural retina (Figures 4(b) and 4(d)). Subsequently, photoreceptor precursor cells expressing specific transcription factor OTX2 appeared, migrated, and accumulated at the apical-most zone of the NR since week 7 (Figures 4(c) and 4(d)). The amacrine and horizontal cells are a class of interneurons found in all vertebrate retinas. AP2 is a transcription factor expressed in the amacrine cells of the developing retina. Likewise, AP2^+^ amacrine cells were found in the intermediate layer of the neural retina on around week 9 and formed a clear nuclear layer on week 13 accordingly (Figure 4(e)). The horizontal cells expressing transcription factor PROX1 also appeared during the similar time window of retinal differentiation (Figure 4(f)). Bipolar and Muller glial cells are late-born cells in retina genesis in vivo. Consistent with that, the urine-derived RPCs differentiated into PKC-*α* ^+^ bipolar cells and CRALBP^+^ Muller cells by W17 (Figures 4(g) and 4(h)). Three U-hiPSC lines tested could repeatedly generate laminated retinal organoids (at least 5 organoids examined from each line, three independent experiments) with all retinal cell types located in the corresponding layer without the need of adding RA into medium like most published studies did at certain time windows [8, 10, 13, 27].

### 3.5. Acquisition of Highly Mature Photoreceptors with Rods and Cones from U-hiPSCs without Addition of Retinoic Acid

**Figure 5:**
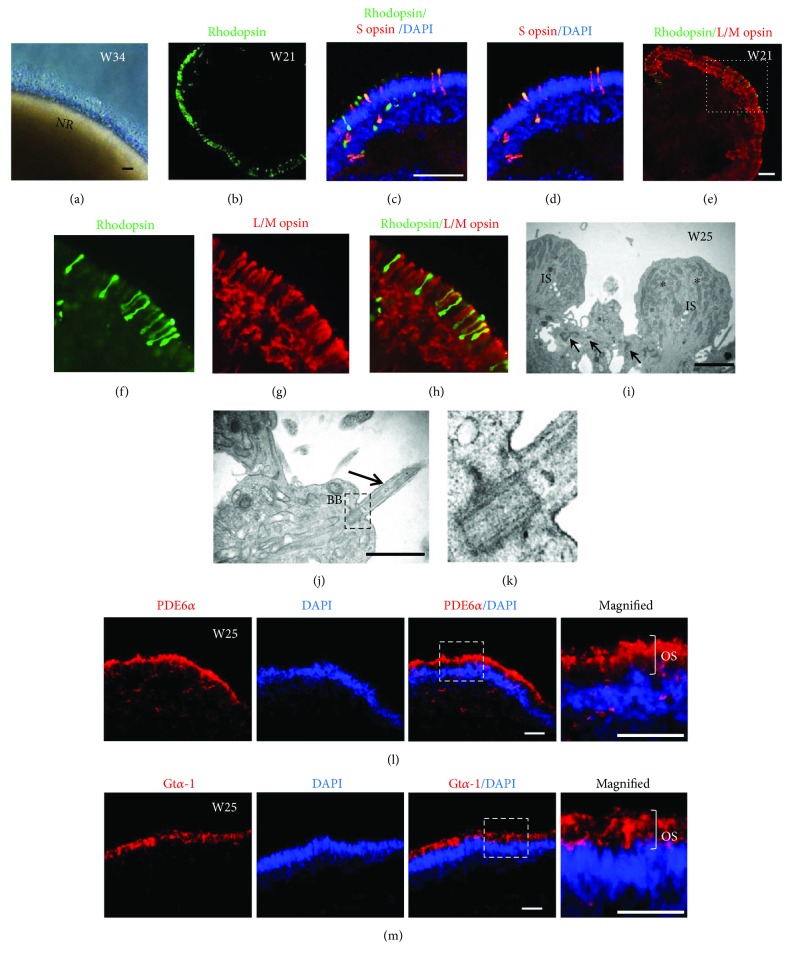
Generation of highly mature photoreceptors with rods and cones from U-hiPSCs without addition of RA. (a) An example image of retinal organoids with long and brush-like segments at apical side. Scale bar = 25 *μ*m. (b–h) Representative images of immunofluorescence staining from UE017 U-hiPSCs showed photoreceptors at W21 differentiated into all subtypes, rhodopsin-positive rods, S opsin-positive blue cones, and L/M opsin-positive red/green cones (b–h). Scale bar = 50 *μ*m. (b) An organoid with rich rod photoreceptors occupying the whole outer nuclear layer (ONL). (c–d) An organoid with a patch of S opsin-positive blue cones and rhodopsin + rods. (e) An organoid with rich cone photoreceptors occupying almost the whole ONL. (f–h) Higher magnification of the squared area in (e). (i–j) TEM revealed that U-hiPSC-derived photoreceptors developed typical ultrastructures, outer limiting membrane (arrow), inner segment (IS) with rich mitochondria (^∗^), connecting cilium (arrow), and basal body (bb). Scale bar = 1 *μ*m. (k) Higher magnification of the squared area in (j). (l, m) U-hiPSC-derived photoreceptors on W25 expressed functional phototransduction proteins PDE6*α* and Gt*α*-1 distributed most in presumptive outer segments (OS). Scale bars = 25 *μ*m.

Many factors influence the specification and maturation of photoreceptors, such as RA [28]. Building upon a previous study showing that reduced concentration and exposure time of RA treatment promoted hiPSC-derived PRC maturation [10], here, we further explored whether the addition of RA is required for PRC development in 3D retinal organoids. To answer this question, we differentiated U-hiPSCs into retinal organoids step by step and kept them for long-term culture without supplement of any RA to the media throughout the whole differentiation process. Consequently, retinal organoids (*n* > 4, per line) derived from U-hiPSCs were kept in a good shape and NR developed long and brush-like segments on the surface since W21 after differentiation (Figure 5(a)). Immunocytochemistry showed that apically located cells expressed markers rhodopsin, L/M opsin, and S opsin, specific for mature photoreceptor subtypes rods, red/green cones, and blue cones, respectively, forming an outer nuclear layer (ONL) as in vivo (Figures 5(b)–5(h)). The rods and cones were well organized with polarized distribution since W21 after differentiation. What is more, using our modified retinal differentiation approach without RA supplement, U-hiPSCs produced not only rod-rich photoreceptors (Figure 5(b)), which was often reported before, but also red/green cone-rich ones (Figures 5(e)–5(h)), which were hardly reported so far. However, the number of blue cones derived from U-hiPSCs did not change much, still with a few or a small patch of S opsin-positive cells per organoid (Figures 5(c) and 5(d)) as reported before [8, 10, 27]. Of all retinal organoids older than W21 assessed (*n* = 14 from three U-hiPSC lines), approximately, 50% (7/14) was rod-rich organoids while 43% (6/14) was cone-rich. Variation existed among organoids and U-hiPSCs lines, consistent with previous reports [14]. In this study, all cone-rich organoids were generated from UE017. More experiments will be needed to clarify these variations.

Furthermore, TEM observation demonstrated that U-hiPSCs-derived photoreceptors developed specific ultrastructures including outer limiting membrane, inner segment rich of mitochondria, basal body, connecting cilium, and rudimentary outer segment (Figures 5(i)–5(k)). Finally, functional proteins involved in phototransduction pathway, *α*-subunit of cGMP-phosphodiesterase (PDE6*α*) and *α*-subunit of rod transducin (Gt*α*-1), were also detected in urine-derived photoreceptors at W25 and located in the developing outer segment (OS) region of the cells (Figures 5(l) and 5(m)). These findings indicated that urine-derived photoreceptors were able to achieve quite high degree of maturation with all phenotypes rich of rod and red/green cones under current differentiation conditions.

## 4. Discussion

Here, we generated 3D retinal organoids containing laminated NR and RPE with urine cell-derived hiPSCs using a modified retinal differentiation protocol without addition of RA. Under the specific differentiation conditions, U-hiPSCs recapitulated all major steps of retinal development in vivo, from eye field, neural retina, and retinal pigment epithelium domains (optic vesicle stage) to 3D retinal organoids (optic cup stage) [29, 30]. The specification and maturation of retinal cells in U-hiPSC-derived retinal organoids strictly followed the spatial-temporal principles as in vivo, consistent with retinal cells from hiPSCs derived from other somatic sources [10]. In addition, our study first demonstrated that U-hiPSCs could produce photoreceptors with mature rods and cones. Predominant red/green cones were also achieved in some retinal organoids older than W21 after differentiation. The red/green cone-rich photoreceptors will be in huge demand for cell therapy of retinal degenerations since human daily activities largely depend on cones' function responsible for color vision and high-resolution central vision [31].

Urine is supposed to be a body waste. Recently, technologies have been developed to reprogram cells collected from urine into hiPSCs, which surprisingly let it become treasure from trash. Thousands of urine cells (UC) can be harvested from 50–200 ml middle stream of the micturition, stored, or expanded for several passages. UCs exhibited the epithelial phenotype and were much easier to be reprogrammed to iPSCs than fibroblasts by circumventing the mesenchymal-to-epithelial transition [24, 32]. In addition, the UCs are easily accessible with noninvasive collection in clinical settings, providing advantages over other somatic cells used in a reprogramming study. hiPSCs from somatic cells hold personal genetic and epigenetic information which do matter on uncovering disease mechanisms. To collect somatic cells for creating iPSCs, patients, in particular their parents and relatives, would be more willing to cooperate and donate their urine without cost and pain, compared to other somatic source. In this study, the U-hiPSCs used were transgenic free and viral free which increased application safety and demonstrated to be capable of generating retinal organoids with all retinal cell types. In the niche of 3D retinal organoids, all retinal cells gradually appeared with retinal ganglion cells first generated, followed by photoreceptor cells, amacrine and horizontal cells, and lastly bipolar and Muller glial cells. The spatial-temporal patterns of retinal development with U-hiPSCs were similar to those with hiPSCs reprogrammed from other somatic cells, such as skin fibroblasts and blood cells [10, 11, 33]. Therefore, U-hiPSCs and their derivatives hold great potential for retinal stem cell therapy and disease modeling for retinal diseases.

Mechanisms regulating photoreceptor fate specification remain elusive. The accumulated evidence from both in vivo and in vitro studies has pointed out that RA plays important role in retinal development and photoreceptor determination [28, 34–36]. Addition of exogenous RA promoted rod differentiation in cultures of dissociated embryonic retinas [36], and in chicken retinal explant, but inhibited cone differentiation and altered the patterning of chicken high acuity area equivalent to human cone-rich macular area [28]. In the other hand, to differentiate hPSCs into retinal cells or photoreceptor cells, most approaches were developed with a supplement of RA (0.5 *μ*M~1 *μ*M) in culture medium for certain periods, yielding rod-rich photoreceptors [7, 8, 10, 13, 14, 26, 27]. Recently, a detail work from Xiufeng Zhong and her colleagues showed that a longer period of high concentration (W7–W17 days, 1 *μ*M) of RA exposure hampered photoreceptor maturation. Furthermore, one study showed that hPSCs produced S-cone-rich photoreceptors under specific differentiation conditions [12]. Based on these pioneer works, here we further optimized the published protocol without addition of any RA to the media throughout the whole differentiation process. As a result, the specification and lamination of U-hiPSCs-derived NRs without RA exposure were similar to those with a shortened period of RA exposures reported before [10]. However, under this no exogenous RA culture condition, U-hiPSCs generated not only retinal organoids with predominant rod photoreceptors (approximately 50%, *n* = 14), but also retinal organoids with predominant red/green cone photoreceptors (approximately 43%, *n* = 14) up to W34 after differentiation, which was not achieved in the previous study with RA supplement [8, 10, 27]. Moreover, we observed that some retinal organoids (at least four organoids observed) contained roughly half and half of rods and red/green cones in a given retinal organoid. While blue cones are still kept in the least number among three subtypes of photoreceptors, corresponding to the in vivo situation of human retina, red/green cones account for 90% of human cone photoreceptors and have the greatest impact on sight in AMD, a common incurable blindness in people older than 50 yrs. Recently, Gonzalez-Cordero et al. isolated and purified L/M opsin GFP cones from hPSC-derived W17–20 retinal organoids by FACS for transplantation, yielding approximately 7% L/M cones [13]. Therefore, further study will be needed to disclose the mechanisms regulating the preferential differentiation of photoreceptor subtypes including the impact of both somatic cell source and RA in order to acquire red/green cone-rich photoreceptors for treatment of maculopathy. Collectively, this optimization of retinal differentiation protocol simplified the tedious, time- and labor-consuming experiments, and our study provided a proof of concept that U-hiPSCs were able to produce not only rod-rich but also red/green cone-rich photoreceptors in 3D retinal organoids, benefiting thousands of patients with retinal degenerative diseases.

In summary, our results demonstrate that hiPSCs reprogrammed from urine cells have the capacity to differentiate towards retinal fate and form 3D retinal organoids consisting of NR attached with RPE in suspension culture conditions. In the niche of retinal organoids, U-hiPSC-derived retinal progenitors generate all major retinal subtypes with six neurons and one Muller glia situated in the proper layer. The specification and maturation of retinal cells from U-hiPSCs follow the spatial-temporal patterns as retinal development in vivo, consistent with retinal cells from other somatic-derived hiPSCs. More importantly, with our modified retinal differentiation protocol without supplement of RA, U-hiPSCs are able to produce highly mature photoreceptors with all subtypes, even predominant red/green cones, which will benefit thousands of patients with AMD. Our results indicate that addition of exogenous RA is not necessary for retinal cell specification with hiPSCs and may influence the selection of photoreceptor phenotypes at least in the niche of retinal organoids. Researches on mechanisms regulating the preferential selection of photoreceptor subtypes differentiated from hPSCs will be needed.

## Figures and Tables

**Table 1:** Primer list.

Genes	Forward	Reverse	Product	TM
			Size (bp)	
OCT4	5′-CGAGCAATTTGCCAAGCTCCTGAA-3′	5′-TCGGGCACTGCAGGAACAAATTC-3′	323	60°C
SOX2	5′-ACCAGCTCGCAGACCTACAT-3′	5′-CCCCCTGAACCTGAAACATA-3′	448	60°C
PAX6	5′-CGGAGTGAATCA GCTCGGTG-3′	5′-CCGCTTATACTGGGCTATTTTGC-3′	301	60°C
LHX2	5′-CAAGATCTCGGACCGCTACT-3′	5′-CCGTGG TCAGCATCTTGTTA-3′	284	60°C
RX	5′-GAATCTCGAAATCTCAGCCC-3′	5′-CTTCACTAATTTGCTCAGGAC-3′	279	60°C
SIX3	5′-CCGGAAGAGTTGTCCATGTT-3′	5′-CGACTCGTGTTTGTTGATGG-3′	171	60°C
SIX6	5′-ATTTGGGACGGCGAACAG AAGACA-3′	5′-ATCCTGGATGGGCAACTCAGATGT-3′	385	60°C
GAPDH	5′-ACCACAGTCCATGCCATCAC-3′	5′-TCCACCACC CTGTTGCTGTA-3′	452	60°C

**(a):** 

Primary antibody	Manufacturer	Cat. number	Dilution	Source
OCT4	Abcam	ab18976	1/250	Rabbit
NANOG	Abcam	ab21624	1/100	Rabbit
SSEA4	Abcam	ab16287	1/100	Mouse
TRA-1-81	Abcam	ab16289	1/100	Mouse
TRA-1-60	Abcam	ab16288	1/100	Mouse
SOX1	Abcam	ab87775	1/200	Rabbit
PAX6	DSHB	3B5	1/50	Mouse
OTX2	Abcam	ab21990	1/500	Rabbit
LHX2	Santa Cruz Biotechnology	sc-81311	1/200	Mouse
SIX3	Rockland	600-401-A26	1/500	Rabbit
MCM2	Abcam	ab4461	1/400	Rabbit
CHX10	Millipore	ab9016	1/200	Sheep
FIBRONECTIN	Abcam	ab2413	1/200	Rabbit
ZO-1	Thermo Fisher	33-9100	1/400	Mouse
BRN3	Santa Cruz Biotechnology	sc-6026	1/200	Goat
AP2	DSHB	3B5a	1/35	Mouse
PROX1	Abcam	ab101851	1/2000	Rabbit
PKC-*α*	Abcam	ab32376	1/2000	Rabbit
CRALBP	Abcam	ab15051	1/500	Mouse
Rhodopsin	Abcam	ab3267	1/200	Mouse
S opsin	Gift from Dr. Jeremy Nathans	/	1/5000	Rabbit
L/M opsin	Gift from Dr. Jeremy Nathans	/	1/5000	Rabbit
Recoverin	Millipore	ab5585	1/500	Rabbit
PDE6*α*	Abcam	ab5665	1/100	Rabbit
Gt*α*-1	Santa Cruz Biotechnology	sc-136143	1/2000	Mouse

**(b):** 

Secondary antibodies	Manufacturer	Cat. number	Dilution
Alexa Fluor 488 Donkey Anti-Goat	Invitrogen	A11055	1/500
Alexa Fluor 555 Donkey Anti-Sheep	Invitrogen	A21436	1/500
Alexa Fluor 488 Donkey Anti-Mouse	Invitrogen	A21202	1/500
Alexa Fluor 555 Donkey Anti-Mouse	Invitrogen	A31570	1/500
Alexa Fluor 488 Donkey Anti-Rabbit	Invitrogen	A21206	1/500
Alexa Fluor 555 Donkey Anti-Rabbit	Invitrogen	A31572	1/500

## Data Availability

The data used to support the findings of this study are available from the corresponding author upon request.

## Supplementary Materials

Supplementary Materials Figure 1: urine-derived hiPSCs used were free of integration of exogenous reprogramming factors and vectors. (a, b) Two plasmid vectors containing reprogramming factors. (c) RT-PCR showed that U-hiPSCs did not express exogenous markers OCT4, SOX2, KLF4, EBNA-1, miR-302-367, SV40T, and ORIP. Pm: plasmid DNA; noRT: negative control without reverse transcriptase.
