# Supplementary material for: Generation of Retinal Organoids with Mature Rods and Cones from Urine-Derived Human Induced Pluripotent Stem Cells
Source: Stem Cells Int. 2018 Jun 13;2018:4968658. doi: 10.1155/2018/4968658 (PMC6020468; doi:10.1155/2018/4968658)
Supplement: Supplementary Materials — Figure 1: urine-derived hiPSCs used were free of integration of exogenous reprogramming factors and vectors. (a, b) Two plasmid vectors containing reprogramming factors. (c) RT-PCR showed that U-hiPSCs did not express exogenous markers OCT4, SOX2, KLF4, EBNA-1, miR-302-367, SV40T, and ORIP. Pm: plasmid DNA; noRT: negative control without reverse transcriptase. [file 4968658.f1.docx]

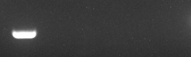

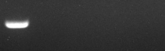

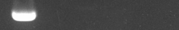

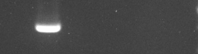

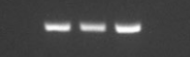

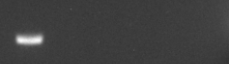

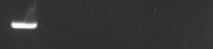

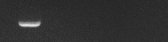


OCT4

SOX2

**pEP4 E02S ET2K**

miR302-367 clusters

OriP

EBNA-1

**pCEP4**

(c)

(a)

SV40LT

OCT4

SOX2

KLF4

SV40LT

ORIP

EBNA-1

Pm UE022 UC005 UE017 noRT

GAPDH

miR-302-367

(b)

KLF4

**Supplementary. Figure 1. Urine-derived hiPSCs used were free of integration of exogenous reprogramming factors and vectors**. (a) and (b): two plasmid vectors containing reprogramming factors. (c) RT-PCR showed that U-hiPSCs did not express exogenous markers OCT4, SOX2, KLF4, EBNA-1, miR-302-367,SV40T,ORIP. Pm: plasmid DNA. noRT: negative control without reverse transcriptase.
